# Cellulose nanofibrils manufactured by various methods with application as paper strength additives

**DOI:** 10.1038/s41598-021-91420-y

**Published:** 2021-06-07

**Authors:** Jinsong Zeng, Zhanting Zeng, Zheng Cheng, Yu Wang, Xiaojun Wang, Bin Wang, Wenhua Gao

**Affiliations:** 1 State Key Laboratory of Pulp and Paper Engineering, Plant Fiber Research Center, South China University of Technology, Guangzhou, 510640 China; 2 China Tobacco Guangdong Industrial Co. Ltd, 88 Huancui South Road, Liwan District, Guangzhou City, Guangdong Province China; 3 School of Chemistry and Chemical Engineering, South China University of Technology, Guangzhou, 510640 China; 4 Guangdong Plant Fiber High-Valued Cleaning Utilization Engineering Technology Research Center, South China University of Technology, Guangzhou, 510640 China

**Keywords:** Materials science, Nanoscience and technology

## Abstract

Recycled paper and some hardwood paper often display poorer mechanical properties, which hinder its practical applications and need to be addressed. In this work, cellulose nanofibrils (CNFs) obtained by a combined process of enzymatic hydrolysis and grinding (EG-CNFs), grinding and microfluidization (GH-CNFs) or TEMPO-mediated oxidation and grinding (TE-CNFs) were characterized by scanning electron microscopy (SEM) and atomic force microscopy (AFM). Moreover, CNFs were made into films on which some characterizations including X-ray diffraction (XRD), Fourier transform infrared spectroscopy (FTIR), and UV–Vis transmittance spectroscopy were implemented. Results showed that CNF fibrillation was promoted as times of passes increased in microfluidization, and CNFs pretreated by enzyme possessed shorter length. Crystallinity of CNFs was related to CNF manufacturing methods, while CNF films’ transparency was correlated to CNF diameter distributions. Moreover, CNFs were applied with different dosages on recycled and hardwood paper. Lengths of CNFs, strength of CNF network, and pulp properties were critical factors affecting the mechanical strength of CNFs-enhanced paper. GH-CNFs showed better strengthened effect on tensile strength of paper than TE-CNFs and EG-CNFs. The best overall improvement was achieved at GH-CNF10 dosage of 5.0 wt% on hardwood paper. The increment of tensile index, burst index, and folding endurance were 108.32%, 104.65%, and 600%, respectively. This work aims to find out the relationship between production methods and morphologies of CNFs and how the morphological characteristics of CNFs affecting the mechanical performance of paper when they are added as strength additives.

## Introduction

Along with the depletion of petroleum resource, the requirement for biodegradable, recyclable, and sustainable substitution is surging quickly. As renewable biopolymer widely existing in the cell walls of the plants as well as animals or bacteria, cellulose is a potential alternative for petroleum-based resource^1^. Cellulose is made up of β-D-glucopyranose connected by β-1-4-linkages with the basic repeating unit named glucose, and the degree of polymerization (DP) of cellulose varies from several hundreds to over ten thousands^2^. Furthermore, cellulose consists of crystalline and amorphous regions^3,4^. Cellulose has a hierarchical structure ranging from micro to nano scale and much attention has been paid to nanocellulose because it is a renewable bio-based material with superb performance in various fields^5^. Nanocellulose can be defined as cellulose whose at least one dimension (length, diameter, or height) is at nanoscale regardless of their source or manufacturing methods^6^. Among all the major categories of nanocellulose, cellulose nanofibrils (CNFs) have always been attractive to the researchers due to its superior properties including large specific surface area, high stiffness, high strength, low weight, high biocompatibility, easy film-forming capability, and therefore can be utilized to fabricate aerogel used as oil absorbent^7^ and cargo carriers on water^8^. CNFs can also act as structural materials and strength additives, for example, lightweight composites in aerospace and automotive^9^. Thus, the applications of CNFs include sensors^10^, food packaging^11^, electrode^12^ and microbatteries^13^.

The production methods of CNFs usually consist of biological, chemical pretreatment, and mechanical disintegration or recombination of them. However, high energy consumption limits the application of mechanical methods^14,15^. Hence, biological, or chemical pretreatments which facilitate subsequent mechanical process are employed in order to reduce energy cost^16,17^. Biological and chemical pretreatment for the preparation of CNFs typically includes enzymatic hydrolysis^18^, TEMPO-mediated oxidation^19^, carboxymethylation^20^, phosphorylation^21^ and etc., while ultrafine grinding^22,23^, homogenization^24^, ultrasonication^25^, and refining^26^ are some of the most prevailing mechanical treatments. Producing CNFs by TEMPO-mediated oxidation are ubiquitous. Carboxyl groups are selectively introduced at C6 of glucose unit and isolation of nanofibers is facilitated^27,28^. Endoglucanases are the most intriguing substance for the enzymatic production of nanocellulose. The principle of endoglucanases reaction is that they make the fibers swell and segregate the internal bonds of the glycan chains in a stochastic way which causes the nonreducing end of the cellooligosaccharides to emerge. Endoglucanases help to facilitate latter mechanical treatment and lower energy consumption^18^. One of the frequently used techniques for producing CNFs from cellulose is ultrafine grinding. Static and rotating grinding stone are equipped with the grinder in which the pulp slurry is passed through. During the grinding process, the hydrogen bond and cell wall structure of the cellulose are broken down by shear forces and most of the pulp is isolated into nano dimensions^29^. The gap between the two grinding stones can be adjusted under different circumstances. Referring to the hydrogen bond, Lindman and colleges asserted that hydrophobic interactions are more important than hydrogen bonding, therefore, are responsible for the stability of cellulose and for its bad solubility in polar solvents^30^. Microfluidizer is an equipment having a similar mechanism as high-pressure homogenizer which is commonly utilized to produce CNFs. Traditionally used in the pharmaceutical field for producing emulsion or other products^9^, microfluidizer is also able to fibrillate the fiber into fibrils. The narrow high-pressure chamber in which the particle undertakes high impact and collides with one another with the purpose of split the fibers off into fibrils is always built in Z-shape or Y-shape^3^.

Although most of the paper is capable of being recycled^31,32^, the recycling rate is rather low which results in abundant resource waste and the generation of greenhouse gases due to inappropriate recycling and treatment^33^. As a result, there is a huge requirement to recycle paper and utilize it properly. Recycled paper is a precious source for paper manufacture with low cost. Because of the decrease in fiber length and decline in tensile strength, in most cases, the quality of the recycled fiber does not meet the need of the end uses of customers^34^. For this reason, chemical additives and fillers are added to improve the performance of the recycled paper. Because sometimes chemical additives need to be utilized with higher dosages, CNFs are better options for being used as strength additives on recycled paper since they are environmental-friendly and biodegradable. CNFs can be potentially applied in large volume in paper and paper board products as strength additives owing to its excellent inherent mechanical strength and high specific surface area^3^. In 1969, the theory of paper tensile strength was first launched out^35^, while in recent years, Taipale, et al.^36^ found that mechanical performance of paper is dependent on the following factors including fiber length, degree of fiber bondability, and strength of the fiber bonds. Some research has been made to investigate the possible applications of CNFs to strengthen paper sheet made by recycled pulp. Campano, et al.^37^ studied the effect of CNF dispersion on properties of the recycled paper sheet. The results demonstrated that with use of dispersing agent at low dosage (0.003%), tensile index still rose at 20.6%. It was reported by another researcher that paper produced by recycled pulp with the addition of CNFs and C-PAM-B or chitosan used as retention agent showed marvelous paper evenness and high mechanical strength^38^. Except for recycled pulp fibers, CNFs has also been utilized to reinforce normal pulp fibers and improvement of different mechanical strength could be found^39,40^. However, much of the previous research focused on CNFs being used with retention agents as a dual system in paper. To the best of our knowledge, few of the papers gave out interpretation of the relationship between the morphological characteristics and the effects of only CNFs utilized as strength additives in different pulps. Furthermore, not much research has been done with CNF dosages of over 5.0 wt%. Moreover, abundant related research was launched with just one single method of CNF production and only a particular pulp was reinforced by CNFs. Few comparisons were made about the morphological variations among different production ways of CNFs. In addition, even less experiments were done to find out how reinforced effects were affected by different species of pulps.

**Figure 1:**
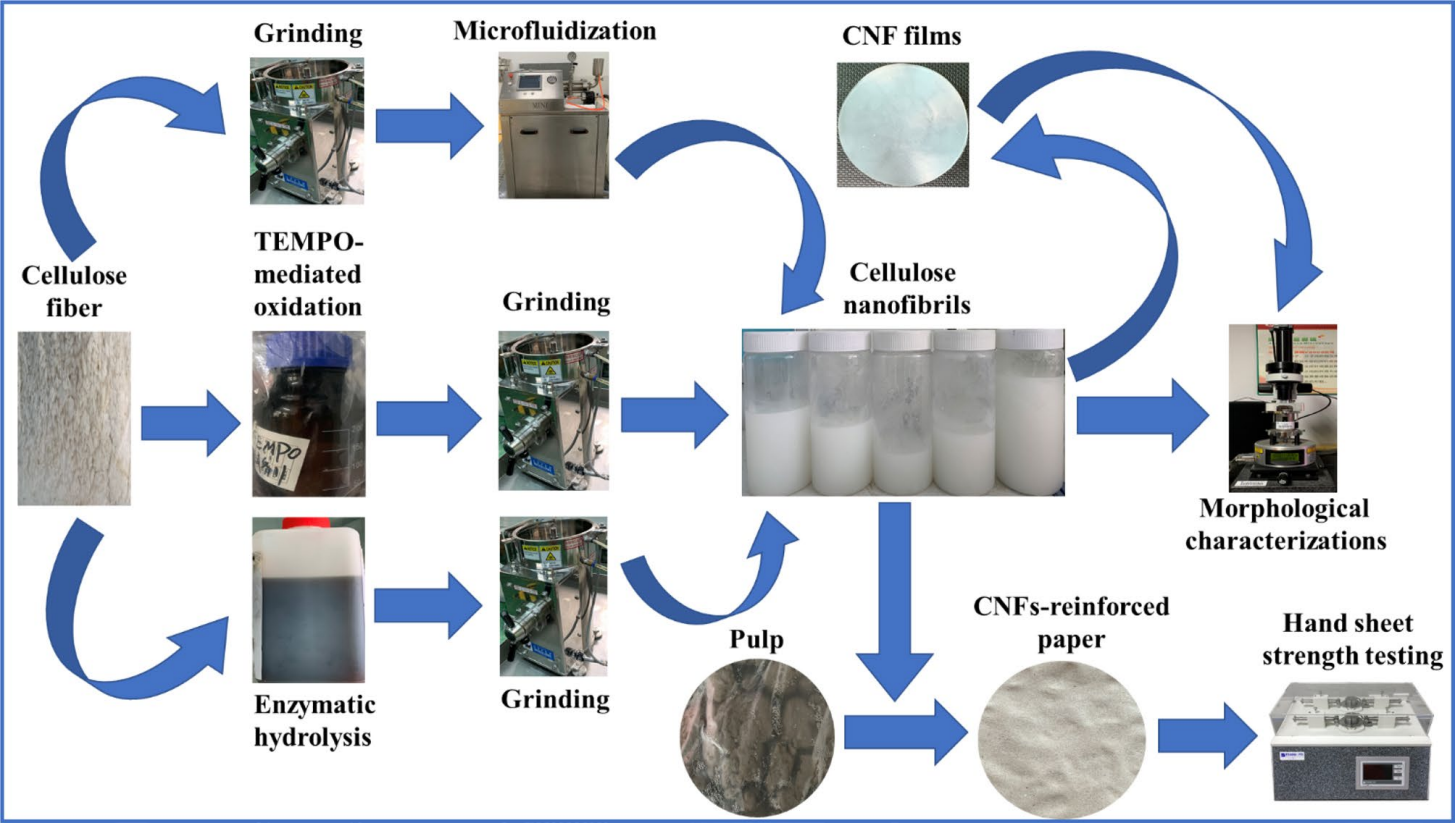
Scheme of detailed operations.

In this paper, we carried out the study on the preparation and morphological characterization of CNFs manufactured from bleached softwood kraft pulp (BSKP) or pine pulp by multiple methods and compared their morphological differences which had impact on their application as strength additives in both recycled paper sheet and hardwood paper sheet. The scheme of detailed operation was shown in Fig. 1. There were three main different CNFs in this work: (1) ones manufactured by grinding and microfluidization (GH-CNFs), (2) ones manufactured by enzymatic treatment and grinding (EG-CNFs), and (3) ones manufactured by TEMPO-mediated oxidation and grinding (TE-CNFs). The aim of this work was to find out the principles of how the morphological traits of CNFs were influenced by different production methods and the mechanisms of how CNF characteristics and different pulps’ properties affecting the mechanical strength of paper.

## Materials and methods

### Materials

The never-dried commercial bleached softwood kraft pulp provided by a paper mill (Guangdong, China) was selected as the source of the CNFs. There were two kinds of recycled pulp in this research. The recycled pulp provided by Nine Dragon paper mill (Guangdong, China) was named as recycled pulp A. The recycled pulp supplied by Huatai paper mill (Jiangmen, China) was named as recycled pulp B. The commercial bleached hardwood pulp which was also used for the preparation of paper was provided by a paper mill (Guangdong, China). The endoglucanase (BANZYME 2900) whose activity was 7.3 IU/mL was purchased from Banmark firm, Finland. The commercial pine pulp was provided by Hubei Chemical Fiber Co., Ltd. (China) and purchased as pulp board directly before use. The 2,2,6,6-Tetramethylpiperidine-1-oxyl (TEMPO, purity ≥ 98%) was provided by Sigma Aldrich (St. Louis, MO, USA).

### Enzymatic treatment

Enzymatic hydrolysis was conducted in a sealed plastic bag at 50 ℃ at neutral condition with a BSKP concentration of 5.0 wt% for 2 h. According to previous research, the dosage of endoglucanase was 15 and 20 mg/g, respectively^41^. To facilitate full contact between BSKP and endoglucanase, the mixture was kneaded every 5 min. As soon as the reaction had finished, boiled water was utilized to inactivate the endoglucanases, and the enzyme treated BSKP fibers were kept over 80 ℃ for 20 min^42^. Then, the flat centrifuge was employed to remove the liquid.

### TEMPO-mediated oxidation

The pine pulp was oxidized by the TEMPO/NaBr/NaClO system under alkaline conditions. 0.5 g freeze-dried cellulose was blended with 0.004 g TEMPO, 0.025 g NaBr, and 500 mL water in a 1000 mL beaker. The TEMPO-mediated oxidation was initiated after the addition of NaClO solution (3 mmol/g cellulose) to the mixture. The pH of the oxidation system was kept constantly at 10.0 by adding 0.5 M NaOH solution. The solution was stirred at 500 r/min for about 1 h and quenched subsequently by adding 10 mL alcohol. Then the suspension was filtered and washed with deionized water until it became neutral.

### Grinding

The BSKP fibers after enzymatic treatment was added to deionized water at a concentration of 1.0 wt% and then stirred for 2 h by a mechanical blender at 1000 rpm to make them fully dispersed. The suspensions were grinded by super mass-colloider (MKCA6-2J, Masuko Sangyo Co. Ltd., Japan) at 2400 rpm. The motion zero position was set as the contact position between two stones before suspension loading. To preclude clogging of the fibers in the grinder, the suspensions were passed through the gap of + 50, + 20, 0, − 20, − 50, − 80 μm for 20 times, respectively. Then the gap between the two grinding disks was adjusted to − 100 μm. The BSKP fibers which were pretreated by endoglucanase with a dosage of 15.0 mg/g or 20.0 mg/g for 2 h and grinded in the way depicted above was named as EG-CNF1 and EG-CNF2, respectively. In addition, some other BSKP fibers that was directly pretreated by 5.0% citric acid and grinded in the same way as EG-CNFs was defined as G-CNFs. For the cellulose suspension, which was oxidized by TEMPO and filtrated, the filtrate was diluted to 2.0 wt%. And then, the suspension was grinded by super mass-colloider (MKCA6-2J, Masuko Sangyo, Japan) at 2500 rpm. The gap between the two grinding disks was gradually adjusted to − 50 μm. The suspension was collected and defined as TE-CNF1 after grinding.

### Microfluidization

The G-CNFs went through microfluidization process by a microfluidizer (MINI, Siemens, Germany) with a concentration of 0.8 wt% at the pressure of 20,000 psi constantly for 8, 10, and 15 times, and they were named as GH-CNF8, GH-CNF10, and GH-CNF15, respectively. The chamber of the microfluidizer known as Z8 had a diameter of 200 μm. The manufacture process of different CNFs was displayed in Table 1.

**Table 1:** Preparation condition of different CNFs.

Sample	Dosage of endoglucanases (mg/g)	Gap between grinding disks (μm)	Passes of microfluidization
GH-CNF8	–	− 100	8
GH-CNF10	–	− 100	10
GH-CNF15	–	− 100	15
EG-CNF1	15.0	–	–
EG-CNF2	20.0	–	–
TE-CNF1	–	− 50	–

### Scanning electron microscopy (SEM)

The scanning electron microscopy (EVO 18, Zeiss, Germany) was utilized to observe and assess the morphology of the CNFs. The accelerating voltage of SEM was 10 kV. And the detector was secondary electron. The samples were dropped onto the mica plate after dilution. And it was coated with a layer of gold in vacuum atmosphere using an ion sputter (Hitachi, MC1000) after drying at room temperature overnight to ensure its electrical conductivity before testing. Thickness of the gold on the samples was around 30 nm. The SEM images were applied to measure the lengths and diameters of the CNFs by using Nano-Measurer 1.2 software. For each CNF sample, one thousand and five hundred fibers from different SEM pictures were used to evaluate their lengths and widths. Lengths and widths of the fibers were measured manually. Lines were carried out along the actual shape of the CNFs to measure their lengths with the utilization of Nano-Measurer 1.2.

### Atomic force microscopy (AFM)

The atomic force microscopy (Nanoscope IIIa, Veeko, USA) was used to characterize the morphology of the CNFs as well. The samples were diluted to a concentration of 0.000005 wt%, then a drop of diluted sample was dispersed on the mica slice and dried at room temperature overnight before testing. The test was performed under the contact mode of the equipment with a tip made of monocrystalline silicon, possessing a radium of 10 nm. The AFM was implemented with a scan rate of 0.977 Hz and scan lines of 512. The post treatment of the AFM images was done on Nanoscope Analysis 1.8 on windows 10 operation system. The section function on the software was utilized to make statistics of the widths of the fibrils. A line was drawn across the fibrils and diameters of the fibrils could be calculated according to number differences. The flatten order of the AFM images was set as 2^nd^ before exported.

### CNF films preparation

The CNF suspension was diluted to 0.2 wt% and dispersed using a magnetic stirrer (85-1, Chijiu, China) at 6000 rpm for 20 min. Then the suspension was vacuum filtrated with a vacuum pump (SHZ-D III, Yuhua, China) at 0.1 MPa with 0.45 μm water-based filter membrane (Newstar, China) to produce CNF films. The CNF films were dried with a heat press machine (HM-380, China) at 105 ℃ for 10 min afterwards. The prepared CNF films were utilized to make Fourier transform infrared spectroscopy (FTIR), X-ray diffraction (XRD), and UV–Vis spectrometer analysis.

### Fourier transform infrared spectroscopy (FTIR) analysis

The surface chemistry of the CNF sample was implemented by Fourier transform infrared spectrophotometer (Nicolet IS50-Nicolet Continuum, Thermo Fisher Scientific) using prepared CNF films. The spectral data was collected at wavenumber ranging from 3700 to 560 cm^−1^.

### X-ray diffraction (XRD)

The crystallinity of the CNF films was measured by diffractometer (X’pert Powder, PANalytical). 2θ scans was implemented from 5° to 60°. The crystallinity indices were calculated utilizing the Segal methods^43^,

1 $$CrI\left( \% \right) = \frac{{I_{200} - I_{am} }}{{I_{200} }} \times 100$$

where *I*_*200*_ is the maximum intensity related to the (200) diffraction lattices at 2θ = 22.5°. *I*_*am*_ is the intensity of the amorphous region at 2θ = 18.5°. Though the Segal methods are widely used, Driemeier and Calligaris stated that the Segal method equates peak height with degree of crystallinity, but that peak area is more representative of the fraction of a material that is crystalline^44^. Although the Segal Crystallinity Index is a little controversial, it is based on an experiment that directly involves the crystals. It is easily performed with data from a powder diffractometer, and it is readily understood. Therefore, despite the objections, the Segal Crystallinity Index continues to be used^45^.

### UV–Vis spectrometer analysis

The transmittance of the prepared CNF films was verified by UV–Vis test that was performed with UV–Vis Spectrophotometer (UV-1900, Shimadzu, Japan) at wavenumber from 350 to 800 nm.

### Hand sheet preparation

The recycled pulp fibers A and B were immersed in water overnight and disintegrated in Valley beater. Afterwards, they were centrifuged to remove extra water. The dried commercial hardwood pulp did not experience a disintegrated process. The recycled pulp fibers A and B, and hardwood pulp fibers mentioned above were used for the preparation of hand sheet. The CNFs were added to the recycled pulp suspension at a consistency ranged from 1.0 to 10.0 wt%. The dosage of GH-CNF8 and GH-CNF10 in recycled pulp A was at consistency of 1.0 wt%, 2.0 wt%, 3.0 wt%, and 5.0 wt%, while the dosage of GH-CNF15 was at concentration of 1.0 wt% and 2.0 wt%. For EG-CNF1 and EG-CNF2, the applied concentration in recycled pulp A was at consistency of 3.0 wt% and 5.0 wt%. The application of GH-CNF8 and GH-CNF10 in recycled pulp B was at consistency of 3.0 wt% and 8.0 wt%, while the dosage of GH-CNF15 was 5.0 wt%. The applied consistency of EG-CNF1 in recycled pulp B was 3.0 wt%, 8.0 wt%, and 10.0 wt%. The concentration of EG-CNF2 used in recycled pulp B was 5.0 wt%, 8.0 wt%, and 10.0 wt%. 3.0 wt%, 5.0 wt%, 8.0 wt%, and 10.0 wt% of TE-CNF1 was blended with recycled pulp B, respectively. The dosage of GH-CNF15 in hardwood pulp was 2.0 wt%, while the dosage of GH-CNF8 and EG-CNF1 was both at concentration of 3.0 wt%, and the applied amount of GH-CNF10, TE-CNF1, and EG-CNF2 was 5.0 wt%. The detailed dosages of different CNFs on various types of pulps were displayed in Table 2. The mixture of different pulp fibers and CNFs was disintegrated at 5000 rpm before forming with the Traditional Sheet Mold. Then, the laboratory formed sheets were pressed and dried in a tablet flatter at 80 ℃ for 10 min.

**Table 2:** Dosage of different CNFs on various types of pulps.

Sample	Dosage on recycled pulp A (wt%)	Dosage on recycled pulp B (wt%)	Dosage on hardwood pulp (wt%)
GH-CNF8	1.0	3.0	3.0
	2.0		
	3.0	8.0	
	5.0		
GH-CNF10	1.0	3.0	5.0
	2.0		
	3.0	8.0	
	5.0		
GH-CNF15	1.0	5.0	2.0
	2.0		
EG-CNF1	3.0	3.0	3.0
		8.0	
	5.0		
		10.0	
EG-CNF2	3.0	5.0	5.0
		8.0	
	5.0		
		10.0	
TE-CNF1	0	3.0	5.0
		5.0	
		8.0	
		10.0	

### Hand sheet strength test

The dried hand sheets were conditioned in the test room with a constant temperature of 23 ℃ and relative humidity of 50% for 24 h. Afterwards, the hand sheets were tested with L&W tensile tester (L&W CE062, Lorentzen & Wettre, Sweden), folding endurance tester (S13505, FRANK-PTI, Germany), and L&W bursting strength tester (L&W CE180, Lorentzen & Wettre, Sweden) under the same condition of temperature and relative humidity. The change in mechanical strength of the CNF-reinforced paper compared with the untreated sample was calculated in the following way:

2 $${\text{Change in Paper Strength }}\left( \% \right) = \frac{{{\text{ Strength of CNF Reinforced Paper}} - {\text{Strength of Untreated Sample }}}}{{\text{Strength of Untreated Sample}}}.$$

For each CNFs-reinforced paper sample, five parallel paper was made. And in each paper sample, six parallel specimens were tested for tensile strength, burst strength, and folding endurance. The number of experiments was 30 in total consequently. The detailed mechanical strength was shown on Figs. 7, 8, Tables 4 and 5.

## Results and discussion

### Morphologies and diameter distributions of CNFs

**Figure 2:**
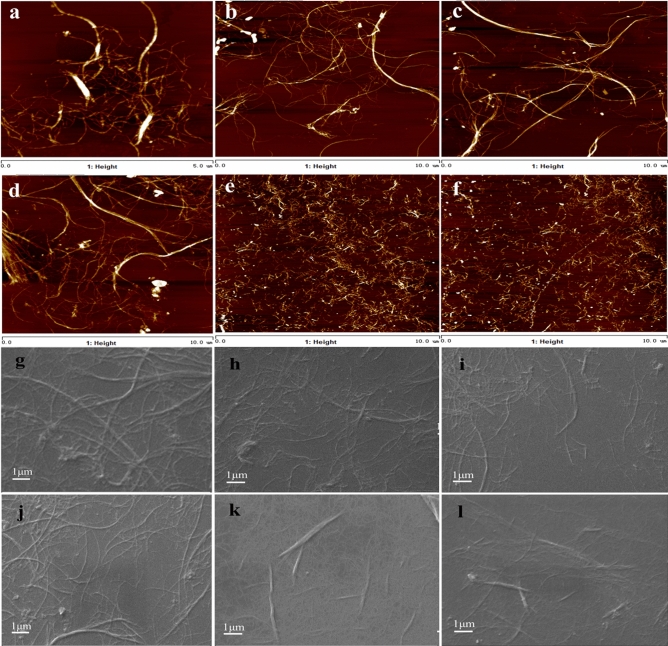
Morphologies of CNFs prepared by different methods. AFM height images: (**a**) EG-CNF1, (**b**) GH-CNF8, (**c**) GH-CNF10, (**d**) GH-CNF15, (**e**) TE-CNF1, (**f**) TE-CNF1; SEM images: (**g**) GH-CNF8, (**h**) GH-CNF8, (**i**) GH-CNF10, (**j**) GH-CNF15, (**k**) EG-CNF1, (**l**) EG-CNF2.

**Figure 3:**
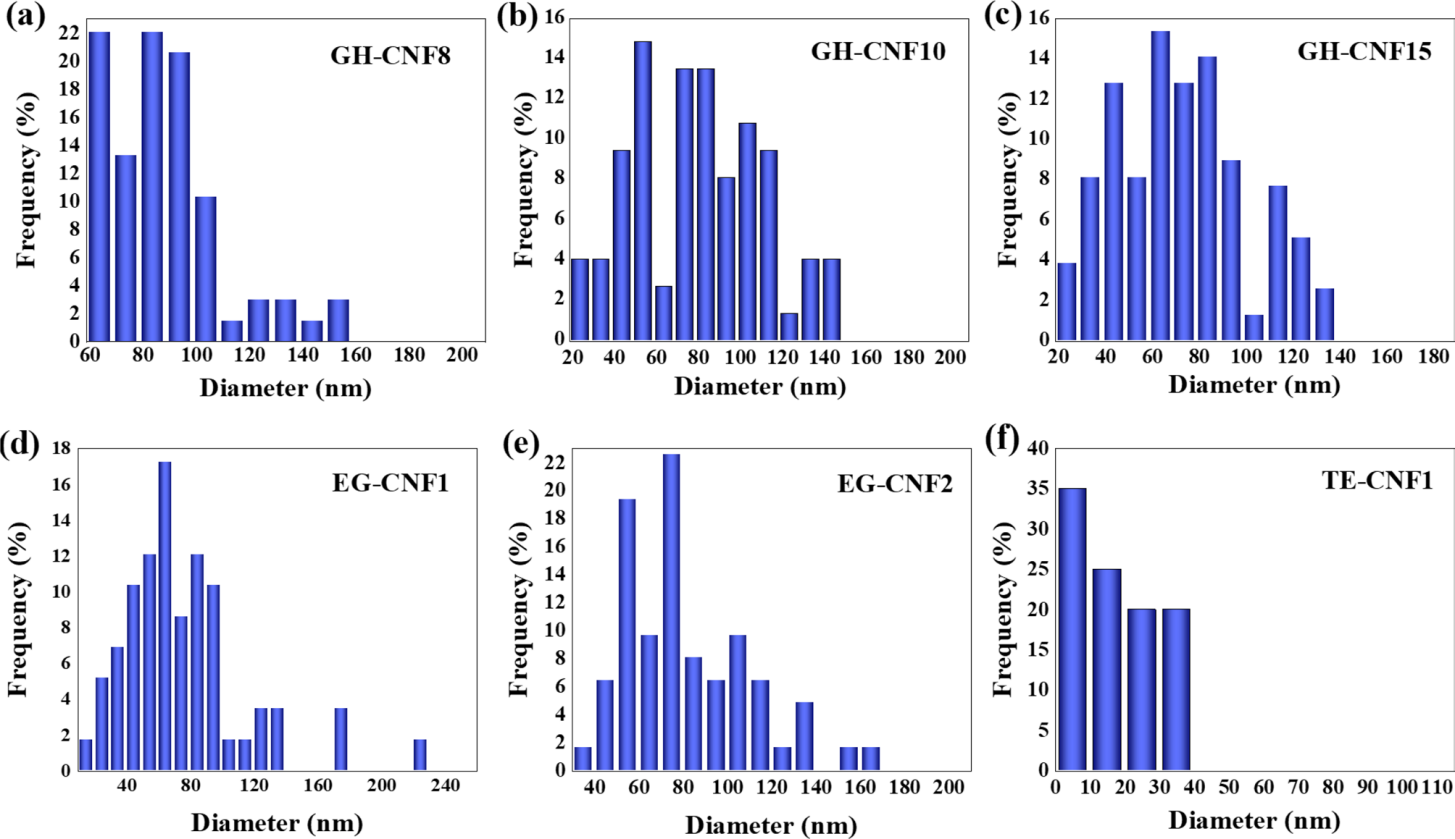
Diameter distributions of CNFs produced by different ways, (**a**) GH-CNF8, (**b**) GH-CNF10, (**c**) GH-CNF15, (**d**) EG-CNF1, (**e**) EG-CNF2, and (**f**) TE-CNF1.

The AFM and SEM images of the CNFs prepared by various kinds of methods are shown in Fig. 2. And the relative concrete implemented parameters of AFM were displayed in Table 3. It is shown that the nanofibrils was delaminated from the fibers and a highly entangled network was formed regardless of the preparing methods. When GH-CNF8, GH-CNF10, and GH-CNF15 are compared, it is easy to figure out that the fiber has been divided into nanoscale and morphology of the CNFs are quite similar because of the application of both grinding and microfluidization. The AFM images shown in Fig. 2e and f and the SEM images displayed in Fig. 2 were used to measure the diameters and lengths of CNFs. The diameter distributions of different CNFs are exhibited in Fig. 3. Results showed that as the pass numbers through microfluidizer increased, the smallest diameter of the CNFs transformed from the interval of 60–70 nm in GH-CNF8 to the interval of 20–30 nm in GH-CNF10 and GH-CNF15. Moreover, CNFs with diameters below 60 nm could not be found in GH-CNF8, while approximate 30% of the GH-CNF10 and GH-CNF15 possessed diameters below 60 nm.

**Table 3:** AFM values of implementation.

Parameter	Value
Scan size	5 or 10 μm
Line direction	Retrace
Scan line	Main
Capture direction	Down
Amplitude setpoint	250.00 mV
Drive amplitude	100.00 mV

This phenomenon was in accordance with the results reported by Lee, et al.^46^. In their study, it was displayed that nanofibrilled cellulose with smaller size and relatively high surface area could be acquired after higher pass numbers. It can be obviously noticed in Fig. 3 that EG-CNF1 had a diameter ranging from 10 to 230 nm and diameter distribution of EG-CNF2 ranged from 30 to 190 nm. The diameter distributions of EG-CNFs were wider than that of the GH-CNFs. This could be interpreted in a way that both ultrafine grinding and microfluidization played a critical role in the individualization of the nanofibrils. Consequently, the GH-CNFs that experienced both ultrafine grinding and microfluidization had a narrower diameter distribution than EG-CNFs which were treated by endoglucanases and went through a totally equivalent grinding process as GH-CNFs. This proved that the number of CNFs with larger diameters would decline after going through the thin chamber of the microfluidizer. Even though endoglucanases did not separate nanofibrils directly, endoglucanases were likely to increase fiber cell wall swelling and the cell walls were delaminated more easily in the latter mechanical treatment^47^. Therefore, it was shown that with the rise in the dosage of endoglucanases, the largest diameter transformed from the interval of between 220 and 230 nm in EG-CNF1 to between 180 and 190 nm in EG-CNF2. In addition, the width of TE-CNF1 ranged from 0 to 40 nm. The small width and narrow diameter distribution of TE-CNF1 certified the capability of pine pulp being defibrillated into CNFs with small widths with the application of TEMPO. The circumstance appeared due to the introduction of negatively charged carboxyl groups and electrostatic repulsion between CNFs. Isolation of CNFs was facilitated in this way^27^. Besbes, et al.^48^ applied Tempo-mediated oxidation for alfa, pine, and eucalyptus fibers at pH = 7 and found that widths of all the samples were between 5 and 20 nm.

### Length distributions of CNFs

**Figure 4:**
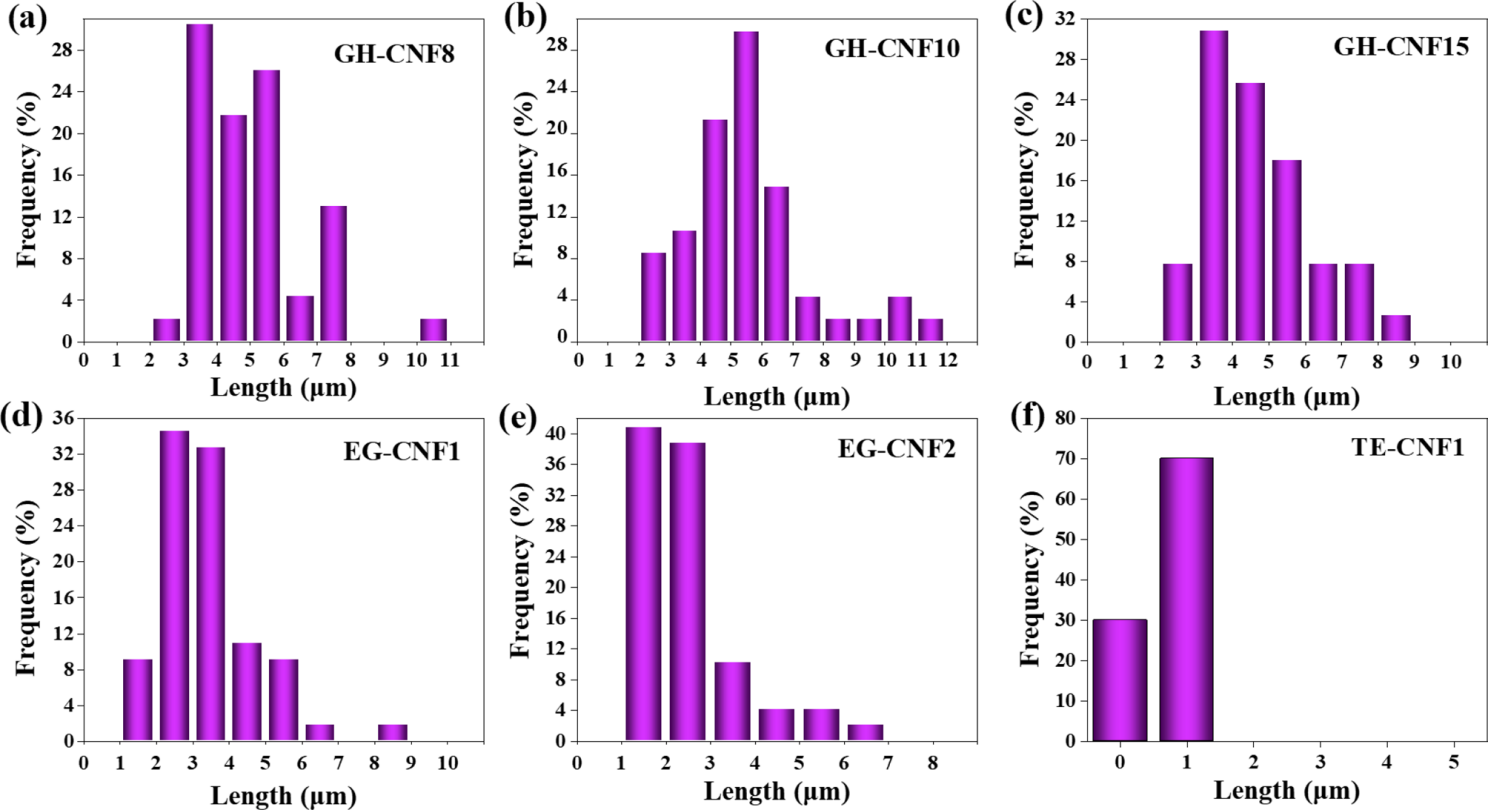
Length distributions of CNFs manufactured by different methods, (**a**) GH-CNF8, (**b**) GH-CNF10, (**c**) GH-CNF15, (**d**) EG-CNF1, (**e**) EG-CNF2, (**f**) TE-CNF1.

According to Fig. 4, the majority of lengths of the GH-CNFs fell into the interval between 3 and 6 μm, while for EG-CNFs, most of the lengths were in the interval between 1 and 4 μm. Therefore, the EG-CNFs pretreated by endoglucanases possessed lower lengths than GH-CNFs in general. This could be further substantiated in AFM images when EG-CNF1 (Fig. 2a) is compared with GH-CNF8, GH-CNF10, and GH-CNF15 (Fig. 2b–d). The circumstance could be explained in a way that endoglucanase can cleave β-1,4 linkages of cellulose chain randomly and hence reduce the lengths of CNFs^18^. In Fig. 4, it was simple to notice that the number of CNFs with lengths of over 5 μm was higher in GH-CNFs than in EG-CNFs. For this circumstance, Nechyporchuk, et al.^49^ reported that during enzymatic hydrolysis, the reduction of the degree of DP for the fibers was usually observed. While Iwamoto, et al.^50^ found out that the DP of fibers decreased when the pass time rose. And furthermore, it was stated that microfluidization process had no harmful impact on the DP of the fibers. Consequently, the EG-CNFs which went through the same grinding process as the GH-CNFs had lower lengths than GH-CNFs. In addition, although differences existed between BSKP fibers and pine pulp fibers, lengths of TE-CNF1 originated from pine pulp were the lowest among all the groups. The phenomenon demonstrated the potential of TE-CNFs possessing smaller lengths than other kinds of CNFs. It occurred because wood cellulose microfibrils in each cellulose fiber was completely converted into separated TEMPO-CNFs due to efficient osmotic effects and electrostatic repulsion among the C6-carboxylated cellulose microfibrils^51^.

### XRD and FT-IR analysis of CNFs

**Figure 5:**
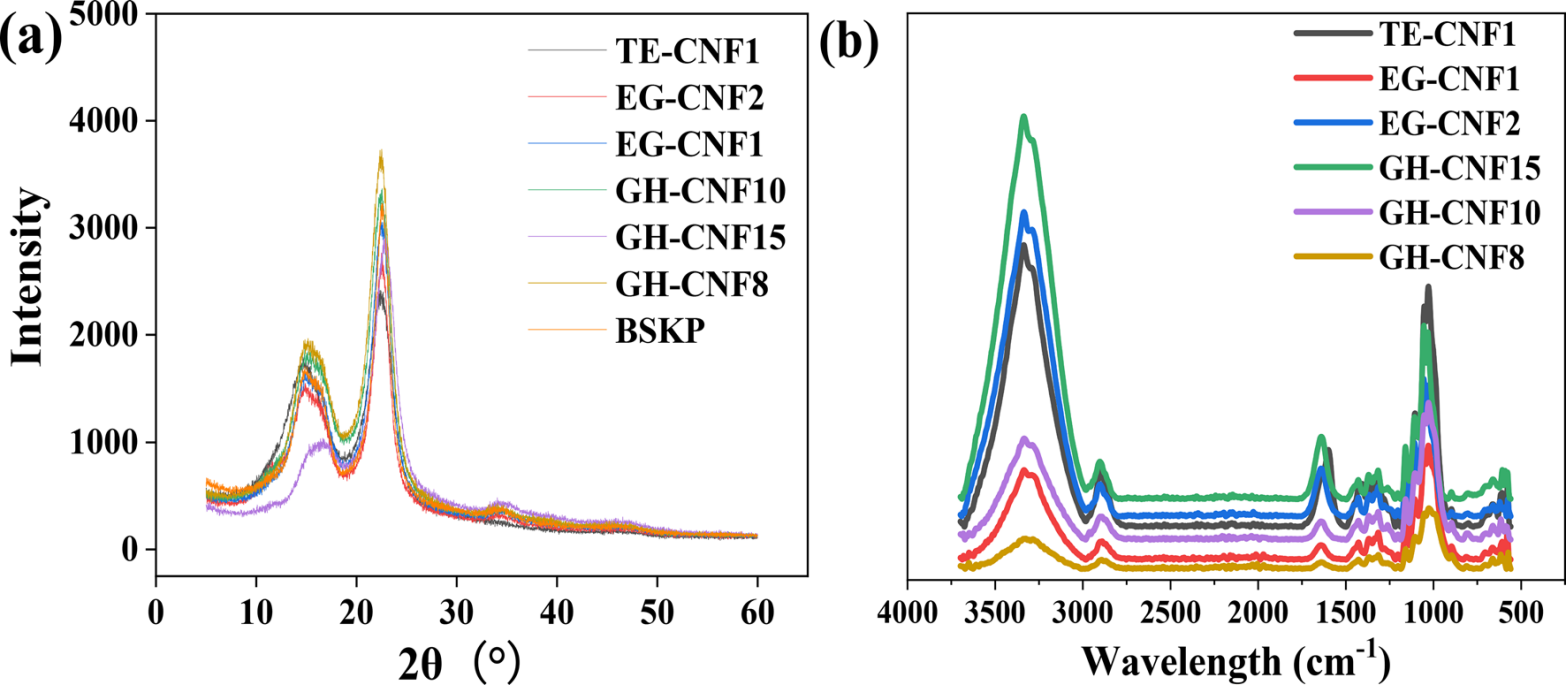
(**a**) XRD spectra of TE-CNF1, EG-CNF1, EG-CNF2, GH-CNF8, GH-CNF10, and GH-CNF15; (**b**) FTIR spectra of GH-CNF8, GH-CNF10, GH-CNF15, EG-CNF1, EG-CNF2, and TE-CNF1.

The XRD spectra of CNFs manufactured by different ways was displayed in Fig. 5a. Crystallinity of the CNF samples was calculated according to Eq. (1). Result showed that crystallinity of BSKP fibers, GH-CNF8, GH-CNF10, GH-CNF15, EG-CNF1, EG-CNF2, and TE-CNF1 was 78.79%, 72.67%, 71.03%, 74.82%, 75.77%, 76.38%, and 66.34%, respectively. The slight decline of crystallinities of GH-CNFs and EG-CNFs compared with their original BSKP fibers could be attributed to the mechanical shearing in grinding process^52^. Since the amorphous region of cellulose was assessed and destroyed by the endoglucanases, and the internal bonds of the glycans was separated^18^, EG-CNFs possessed the highest crystallinity among the six samples. Moreover, the crystallinity of GH-CNF15 was higher than that of GH-CNF8 and GH-CNF10, which was in accordance with the research of Qua et al.^53^. They reported that the crystallinity of samples increased as pass numbers increased in microfluidization because of the removal of amorphous region. In addition, the crystallinity of TE-CNF1 was relatively low which could be interpreted in a way that the oxidation of hydroxyl groups on C6 carbon atoms often leads to a reduction of crystallinity of resultant CNFs^54^. The overlay FT-IR spectrum of CNF was shown in Fig. 5b. Several peaks could be easily noticed in different CNFs. The peak found within 3335–3338 cm^−1^ was related to O–H stretching. And the peak found within 2896–2902 cm^−1^ had a relationship with C–H stretching. Moreover, the peak seen at 1019–1029 cm^−1^ was assigned to C–O stretching^55^. What should be paid attention to is that there was a difference between TE-CNF1 and the other samples. The peaks observed at 1592 and 1603 cm^−1^ were assigned to COO^−^ stretching^52^.

### UV–Vis transmittance measurement analysis of CNF films

**Figure 6:**
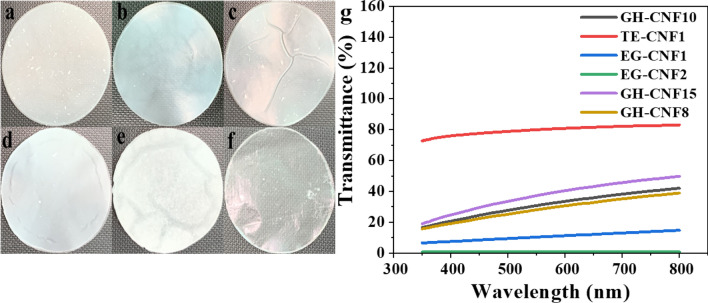
Profile of prepared CNF films. (**a**) GH-CNF8, (**b**) GH-CNF10, (**c**) GH-CNF15, (**d**) EG-CNF1, (**e**) EG-CNF2, (**f**) TE-CNF1, and (**g**) UV spectra of TE-CNF1, GH-CNF15, GH-CNF10, GH-CNF8, EG-CNF1, and EG-CNF2.

As seen in Fig. 6a–f, there is significant difference among the transparency of CNF films made by different measures**.** The transparency of the CNF films is related to their transmittance. In addition, the transmittance of different CNF films at wavelength of 600 nm are used to compare with one another normally^56^. According to Fig. 6g, transmittance of TE-CNF1, GH-CNF15, GH-CNF10, GH-CNF8, EG-CNF1, and EG-CNF2 films at wavelength of 600 nm was 80.81%, 30.61%, 33.52%, 40.41%, 11.23%, and 0.71%, respectively. In fact, the transmittance of different CNF films reflects width of CNFs. The TE-CNF1 whose diameters were below 40 nm got much higher transparency than the other groups. As mentioned above, the degree of fibrillation was enhanced as times of passes increased, therefore, GH-CNF15 displayed better transparency than GH-CNF8 and GH-CNF10. Moreover, EG-CNF1 and EG-CNF2 possessing higher diameter and wider diameter distributions showed lowest transmittance among all the samples. A plausible explanation was that the number of lights scattered rose dramatically when the diameters of nanocellulose became larger and optical transmittance declined consequently^57^. Tarrés, et al.^58^ obtained a similar result that films pretreated by enzymatic treatment had lower transmittance than those films fabricated by TEMPO-mediated oxidation as an impact of diameter distributions.

### Tensile index of CNFs-reinforced paper

**Figure 7:**
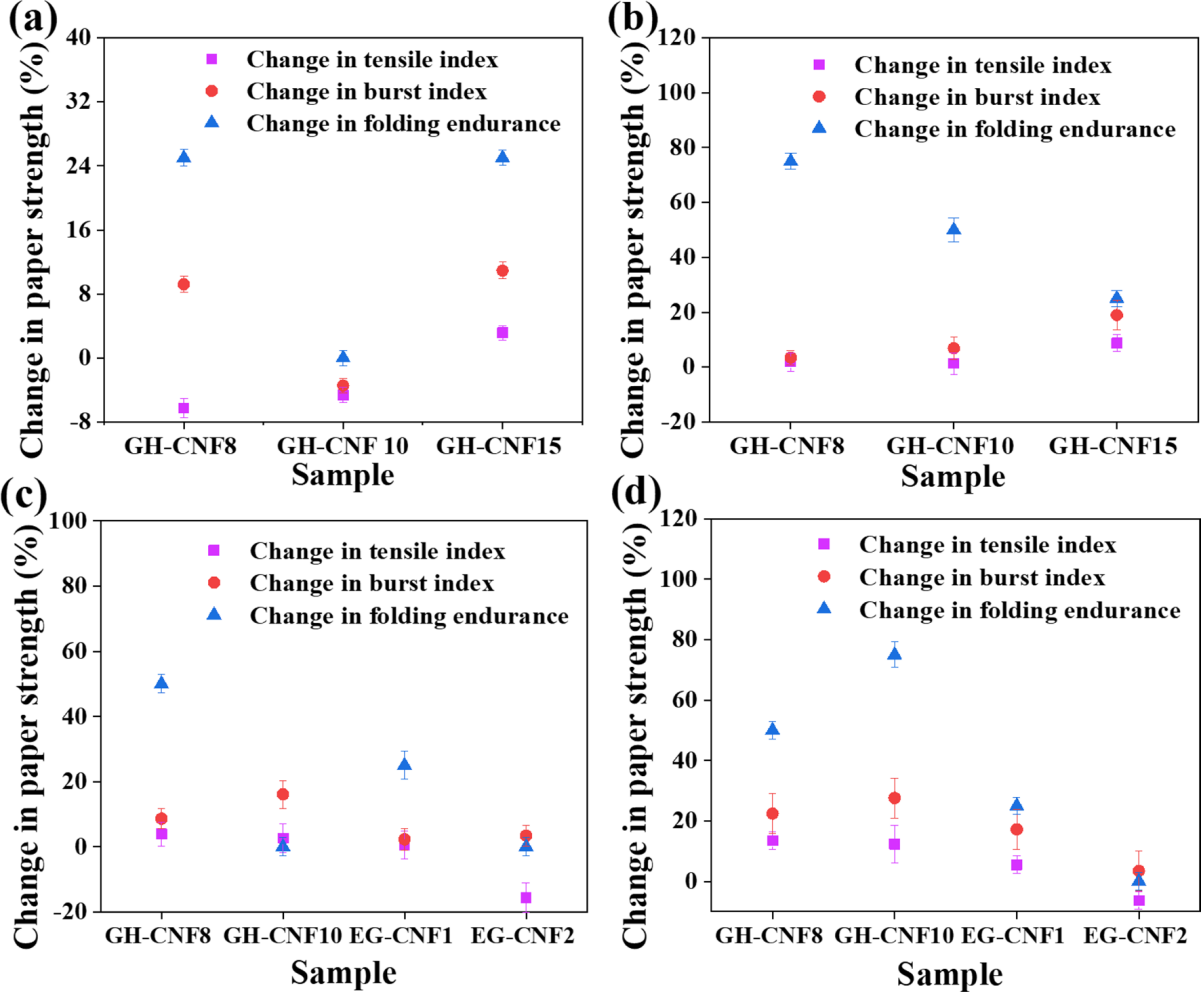
Change in mechanical strength of papers from recycled pulp A strengthened with different CNFs at dosage of (**a**) 1.0 wt%, (**b**) 2.0 wt%, (**c**) 3.0 wt%, and (**d**) 5.0 wt%. All the numbers were normally distributed. The points shown on the figure were average numbers of all the tested specimens and the error bars displayed the standard deviation of all the numbers acquired through testing.

The change in tensile strength of CNFs-reinforced recycled paper A, CNFs-reinforced recycled paper B, and CNFs-reinforced hardwood paper was calculated according to Eq. (2). Compared with the untreated recycled pulp sheet, the tensile index of recycled pulp sheet with supplement of 1.0 wt% GH-CNF8 or 1.0 wt% GH-CNF10 decreased. A reasonable interpretation was that the retention of CNFs was extremely low because most of them flowed away along with the water during the forming section of paper at lower addition of CNFs^59^. As confirmed in Fig. 7, the tensile index of the CNFs-reinforced recycled pulp sheet A rose at higher dosage of 2.0 wt%, 3.0 wt%, and 5.0 wt% in GH-CNF8 and GH-CNF10 compared with the untreated recycled pulp sheet. In addition, as seen in Table 4, the tensile index of recycled paper B reinforced by GH-CNF8, GH-CNF10, and EG-CNF1 was also higher as dosage increased. A plausible explanation for these increments was that the strength of the fiber network was improved by increased mechanical entanglement between CNFs and recycled pulp fibers^60^. Furthermore, more compact structure and higher density of paper sheet was obtained with increase of CNF supplement. And Tajik et al.^40^ has reported that the bond formation between the fibers and fine particles by CNFs was observed with addition of CNFs in paper making process. The mechanism of CNFs used as strength additives to strengthen paper is as follow. The tensile performance of CNF is prominent compared to fibers^61^, and a strong affinity is expected between CNFs and fibers due to the similar structure between them. As a matter of fact, the prerequisite of hydrogen bond formation is that the distance between neighboring hydroxyl groups is less than 0.35 nm. However, distances between fibers are usually not close enough for the bond formation. CNFs play roles as adhesion promoter and bridges, connecting the adjacent fibers with the contribution of their length of several micrometers and entangled network constituted by the cellulose fibrils with nano scale of diameters. As a result, the bonded area between fibers rise and stabler bonding is achieved. Furthermore, the CNFs embedded among the bigger fibers make contribution to the capacity of load bearing of the paper^62^. Consequently, it could be witnessed in Table 4 and Fig. 7 that as the dosage of GH-CNF8 and GH-CNF10 increased, there was an increment in the tensile index of the CNFs-reinforced recycled paper because more bonding was formed between fibers.

**Table 4:** Change in mechanical strength of papers from recycled pulp B strengthened by various kinds of CNFs at different dosages. (For each value, a total number of 30 parallel experiments were done and the numbers shown were mean values).

Sample	CNF dosage	Tensile index (Nm/g)	Tensile index increment	Burst index (Kpa·m^2^/g)	Burst index increment	Folding endurance	Folding endurance increment
Blank	0	18.43	0	1.32	0	2	0
GH-CNF8	3.0%	26.33	+ 42.86%	1.56	+ 18.18%	2	0
GH-CNF8	8.0%	28.96	+ 57.14%	1.93	+ 46.21%	3	+ 50%
GH-CNF10	3.0%	24.07	+ 30.60%	1.37	+ 3.78%	2	0
GH-CNF10	8.0%	29.88	+ 62.00%	1.90	+ 43.94%	2	0
GH-CNF15	5.0%	23.32	+ 26.53%	1.50	+ 13.64%	2	0
EG-CNF1	3.0%	22.75	+ 23.44%	1.41	+ 6.81%	2	0
EG-CNF1	8.0%	25.38	+ 37.71%	1.56	+ 18.18%	3	+ 50%
EG-CNF1	10.0%	27.26	+ 47.91%	1.43	+ 8.33%	4	+ 100%
EG-CNF2	5.0%	23.91	+ 29.73%	1.39	+ 5.30%	2	0
EG-CNF2	8.0%	24.45	+ 32.66%	1.47	+ 11.36%	2	0
EG-CNF2	10.0%	24.07	+ 30.60%	1.41	+ 6.82%	2	0
TE-CNF1	3.0%	24.42	+ 32.50%	1.45	+ 9.85%	2	0
TE-CNF1	5.0%	24.73	+ 34.18%	1.45	+ 9.85%	2	0
TE-CNF1	8.0%	28.09	+ 52.41%	1.67	+ 26.52%	3	+ 50%
TE-CNF1	10.0%	24.99	+ 35.59%	1.58	+ 19.70%	3	+ 50%

It was reported that the improvement effects of CNFs rely on a couple of factors such as the dosage of CNFs and the degree of fibrillation that is related to the specific surface area of the CNFs^63^. Although it was proposed that the mechanical properties can be improved with CNF dosage of between just 1.0 wt% to 10.0 wt%^62^, in some of the research, CNF dosage just reached upper limitation of 5.0% with use of retention agents^39,40^ as higher dosage of CNFs might result in drainage problems. As seen in Table 4, 10.2% increase of tensile index increment of recycled paper B was seen when the dosage of EG-CNF1 increased from 8.0 to 10.0 wt%. A 18.23% rise of tensile index increment of recycled paper B was also seen as dosage of TE-CNF1 changed from 5.0 to 8.0 wt%. In addition, tensile index increment of recycled paper B beyond 57% could be witnessed with usage of GH-CNF8 and GH-CNF10 at dosage of 8.0 wt%. These data certified the potential of using CNFs at dosage of more than 5.0 wt% as strength additives for recycled paper. However, in certain situation, the tensile index increment of recycled paper B reinforced by TE-CNF1 dropped from 52.41 to 35.59% as the dosage of TE-CNF1 transformed from 8.0 to 10.0 wt% as seen in Table 4. The circumstance occurred probably because the space between the recycled pulp fibers was limited. The space could not hold more CNFs in when it was filled up with CNFs. And as a result, fewer enhancement could be seen even when more CNFs were utilized.

### Effect of CNF length on CNFs-reinforced paper strength

**Figure 8:**
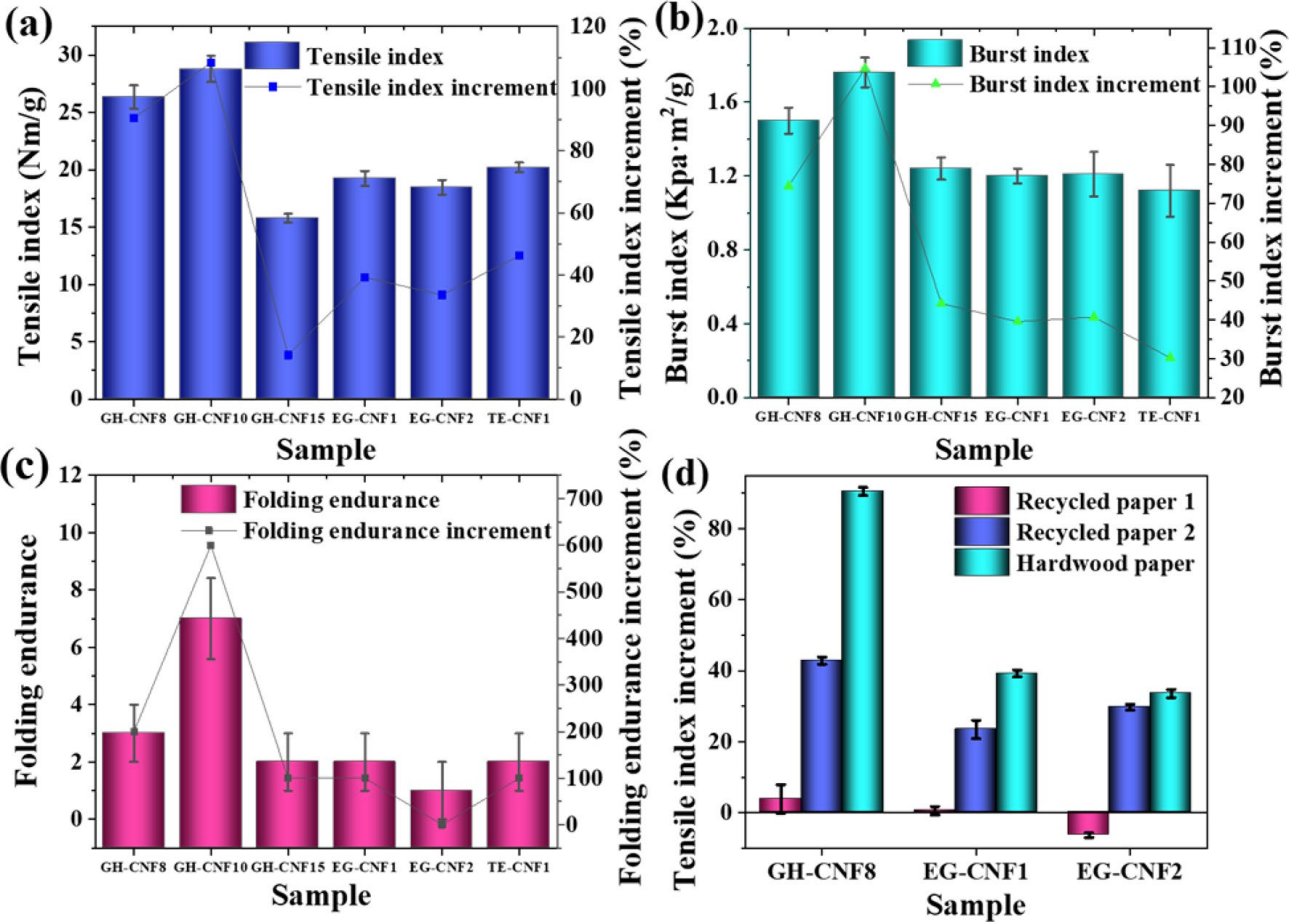
Mechanical strength increment of CNFs-reinforced hardwood paper and comparison of CNF reinforcement on different paper. (**a**) Tensile index increment of CNFs-reinforced hardwood paper enhanced by GH-CNF8, GH-CNF10, GH-CNF15, EG-CNF1, EG-CNF2, and TE-CNF1 at dosage of 3.0 wt%, 5.0 wt%, 2.0 wt%, 3.0 wt%, 5.0 wt%, 5.0 wt%; (**b**) Burst index increment of CNFs-reinforced hardwood paper enhanced by GH-CNF8, GH-CNF10, GH-CNF15, EG-CNF1, EG-CNF2, and TE-CNF1 at dosage of 3.0 wt%, 5.0 wt%, 2.0 wt%, 3.0 wt%, 5.0 wt%, 5.0 wt%; (**c**) Folding endurance increment of CNFs-reinforced hardwood paper enhanced by GH-CNF8, GH-CNF10, GH-CNF15, EG-CNF1, EG-CNF2, and TE-CNF1 at dosage of 3.0 wt%, 5.0 wt%, 2.0 wt%, 3.0 wt%, 5.0 wt%, 5.0 wt%; (**d**) A comparison of tensile index increment on different paper reinforced by GH-CNF8, EG-CNF1, and EG-CNF2 at dosage of 3.0 wt%, 3.0 wt%, and 5.0 wt%. All the numbers were normally distributed. All the bins that have been tested were utilized in the histogram. The plots reported an average number. And the error bars displayed the standard deviation of all the numbers acquired through testing.

It is proposed herein that the length of the CNFs is a critical factor affecting its enhancement effect on paper when CNFs are used as strength additives. As mentioned before, one of the factors that affect the mechanical strength of paper is fiber length^36,64^. As referred to previously, CNFs link the adjacent fibers together as a bridge, but if the CNFs are not long enough to reach the distance between the two contiguous fibers, they will fail to be retained during the water drainage or remain in the recycled fiber matrix without making a difference. This was verified by both Fig. 4 and Table 4. The EG-CNF 2 with most lengths falling into the interval between 2 and 4 μm had a shorter average length than the other four kinds of CNFs and did not show any strengthened effects on recycled paper A with dosage of up to 5.0 wt%. However, the different pulps which were enhanced played a role in the strengthen effect as well. The EG-CNF2 that did not display any enhancement on recycled pulp A exhibited more than 30% improvement to both recycled pulp B and hardwood pulp as confirmed by both Table 4 and Fig. 8a. Paper became denser when CNFs were utilized^15,62^, but there could be differences in density of the fibers in different pulps which caused the situation that CNFs possessed smaller length could not connect the contiguous fibers when fibrous density was low. Similarly, the tensile index of the CNFs-reinforced recycled paper A dropped compared to the untreated sample in the circumstances when just 1.0 wt% of CNFs were added in GH-CNF8 and GH-CNF10. The EG-CNFs with small length and the GH-CNFs with limited dosage were probably loss or retained in the recycled fiber network without playing a role which has led to the uneven formation and the decrease of tensile strength of the CNFs-reinforced recycled paper. As confirmed in Fig. 4, the average length of EG-CNF1 was lower than that of GH-CNF8’s. Therefore, although the lengths of EG-CNF1 were long enough to attach the fibers together and EG-CNF1 were embedded in the fiber network, it still showed lower enhancement effect than GH-CNF8 among recycled paper A, recycled paper B, and hardwood paper upon the same dosage of 3.0 wt% as confirmed in Fig. 8d. As shown in Fig. 7, GH-CNF10 reinforced recycled paper A displayed the optimal mechanical strength increments among all the groups at dosage of 5.0 wt%, with 12.37%, 22.41%, and 75% for tensile index, burst index, and folding endurance, respectively. Moreover, it could be noticed in Table 4 that GH-CNF10 exhibited better reinforcement effect about tensile index on recycled pulp B than the other categories of CNFs with a 62% increment. The largest lengths of the GH-CNF10 reached over 10 μm which were larger than the other four groups. So, the truth that CNFs with higher lengths displayed better strengthened effect on paper certified the importance of the lengths of CNFs on reinforcing strength of recycled paper as well.

### Effect of CNF network on CNFs-reinforced paper strength

According to Table 4 and Fig. 4, even though TEMPO-mediated oxidized CNFs owned small lengths, it still showed similar strengthened effect as other kinds of CNFs when the same dosages of CNFs were used. For example, the tensile index increment of TE-CNF1-reinforced recycled paper B was 9.06% higher than that of EG-CNF1- reinforced recycled paper B at dosage of 3.0 wt%. And upon the same dosage of 8.0 wt%, the tensile index increment of TE-CNF1 reinforced recycled paper B was just 4.73% lower than that of recycled paper B reinforced by GH-CNF8. These phenomena implied that CNF length was not the only factor affecting the strength of CNF-reinforced paper. What is different between TEMPO-mediated oxidized CNFs (TOCNFs) and other CNFs is that TOCNFs are fully individualized fibers with narrow diameter distribution^65^. There is electrostatic repulsion between the nanofibrils^49^, which facilitates the dispersion of the TOCNFs. Therefore, the TOCNFs could be evenly fixed among the networks of the recycled pulp fibers, and paper formation evenness and tensile strength were enhanced consequently. Since carboxylate groups could be found on surfaces of TOCNFs, the TOCNF elements formed self-assemble and nematic-ordered structures in water through electric repulsion working between TOCNF elements^66^. The TOCNF network with uniform orientation made contribution to the high strength of the TE-CNF1-reinforced recycled paper.

### Effect of original pulp on CNFs-reinforced paper strength

Djafari Petroudy, et al.^39^ utilized 5.0 wt% CNF which was prepared by refining and homogenization to produce paper with bagasse pulp and a 38.8% increase of tensile index was acquired compared with the untreated sample. In the current research, 3.0 wt% of GH-CNF8 and 5.0 wt% of GH-CNF10 manufactured by grinding and microfluidization were utilized to make paper with hardwood pulp, as a result, tensile index increment of CNFs-reinforced paper reached over 90%. Delgado-Aguilar, et al.^67^ utilized CNFs from TEMPO-oxidized bleached fibers to enhance recycled paper and found that high tensile strength could be achieved at dosage of 1.5%. An apparent increase of tensile index of CNFs-reinforced recycled paper B was reported here at dosage of 3.0wt%.

Actually, differences in the manufacturing methods might affect properties of the CNFs and have a further impact on the performance of CNFs utilized as strength additives. However, the original pulp also played a part on CNF strengthened effect. Delgado-Aguilar, et al.^67^ stated that the recycling process has detrimental influence on the recycled pulp, including the reduction in inter-fiber bonding and mechanical properties and fiber was shortened during the recycling process^34^. Hence, refining was presented to reinforce the tensile strength of recycled paper A and B while hardwood pulp did not experience the same process. There were differences on tensile index of the paper made from these three kinds of pulps without CNFs added consequently. Recycled paper A presented the highest tensile index of 25.07 Nm/g followed by recycled paper B of 18.43 Nm/g and tensile index of hardwood paper was the lowest among three of 13.82 Nm/g. Based on Fig. 8d, the tensile index increment was higher on hardwood paper than recycled paper A and recycled paper B at the same dosage of GH-CNF8, EG-CNF1, and EG-CNF2. One of the important reasons the situation occurred was that both recycled pulp A and recycled pulp B went through mechanical refining process which caused fines in the pulps. Moreover, the potential of CNFs playing as bridges between fibers was hampered in a way as the recycled pulps’ fibrillation were facilitated in the refining stages^62^. Another reason that GH-CNF8, EG-CNF1, and EG-CNF2 manifested lower tensile index increment on recycled paper A and recycled paper B than hardwood paper was as the fibers were recycled, the recycled pulp displayed lower conformability and the phenomenon of hornification^67^. Consequently, the inter-fiber bonding ability between CNFs and fibers was compromised. Furthermore, it is known that fibers were shortened^68^, and defects of fibers appeared during the recycling process. As the recycling times and conditions might vary, there were probably differences on strength of recycled fibers A and recycled fibers B that caused the different enhancement effect about tensile index of the recycled paper A and recycled paper B using the identical CNFs upon same dosages according to Fig. 7 and Table 4.

### Burst index of CNFs-reinforced paper

The change in burst strength (burst index increment) of CNFs-reinforced recycled paper A, recycled paper B, and hardwood paper was calculated based on Eq. (2). The change in burst index of different paper enhanced by various kinds of CNFs had a different trend compared with the change of tensile index. Value of the burst index reflects the highest vertical pressure paper can resist before rupture^67^. The CNFs supplied to the recycled pulp and hardwood pulp caused a denser network^40^, which led to the improvement of bonding formation between fibers and the enhancement of burst strength of the CNFs-reinforced paper^69^. As reflected in Fig. 8b, the burst index of paper reinforced by different kinds of CNFs showed an increment ranging from 30.23 to 104.65% compared with the untreated sample. However, in the groups of recycled paper A reinforced by GH-CNF8, GH-CNF10, and EG-CNF2, the burst index emerged in a stochastic way without an obvious order. A similar transformation trend of burst index could be also observed on recycled paper B enhanced by EG-CNF1, EG-CNF2, and TE-CNF1. As seen in Table 4, burst index increment dropped after increasing when the dosage of EG-CNF1 transformed from 3.0 to 10.0 wt%, TE-CNF1 from 3.0 to 10.0 wt%, and EG-CNF2 from 5.0 to 10.0 wt%. It was narrated that the additives could possibly have positive or negative impact on the mechanical performance of the paper because of the special strengthening behavior of the additives^70^. What could be noticed in Table 4 was that burst index increments were higher on hardwood papers than the other two kinds of recycled papers upon same dosages of identical CNFs. When CNFs are utilized in paper making, they fill in the matrix of the pulp fiber and decrease of air permeability is noted^15^ regardless of the pulp utilized. One of the plausible reasons that differences of burst index increments existed was that refining process was implemented on both recycled pulp A and B. Fibers became more flexible and bonding abilities were improved during the refining process, therefore, a highly fibrillated network which was benefit for resisting pressure had been made before CNFs were added. In other words, CNFs had the potential to make larger difference on presenting higher density for hardwood paper. Another reason was the same as what was seen in tensile index increments. More rigid fibers of recycled pulp led to weaker bonding between CNFs and recycled fibers^67^.

### Folding endurance of CNFs-reinforced paper

The folding endurance increments of CNFs-reinforced paper was calculated according to Eq. (2). Unlike tensile index and burst index, folding endurance increments relied more on the categories of the original pulps. Based on Fig. 7, recycled paper A reinforced by CNFs showed a folding endurance increments between 25 and 75%, while the majority of recycled paper B did not display folding endurance increments with CNFs utilized. And folding endurance increments equal to or more than 100% were recorded for most of the CNFs-reinforced hardwood paper as confirmed by both Fig. 8c and Table 5. Folding endurance reflects the flexibility and plasticity of the paper. The complexity of the CNFs falling into the fiber matrix and affecting the bonding may have resulted in the complicated results of the paper folding endurance enhanced by CNFs. Another possible interpretation was that the drainage of the water from a certain height led to the random fiber orientation of each group of paper. Higher folding endurance increment implicated that the fibers of the CNFs-reinforced hardwood paper was more capable of remaining the original orientation and flexibility^37^. Moreover, the folding endurance testing was performed in a small part of the paper. The test is sensitive to the changes of the paper, consequently, there will be tremendous difference when a small change is observed in the formation of the paper. The folding endurance can also be affected by factors like the drying temperature and thickness of paper^71^.

**Table 5:** Comparison of burst index and folding endurance increment of different papers enhanced by identical CNFs of same dosages. (For each value, a total number of 30 parallel experiments were done and the numbers shown were mean values).

Sample	Dosage (%)	Recycled paper A burst index increment (%)	Recycled paper B burst index increment (%)	Hardwood paper burst index increment (%)	Recycled paper A folding endurance increment (%)	Recycled paper B folding endurance increment (%)	Hardwood paper folding endurance increment (%)
GH-CNF8	3.0	+ 8.62	+18.18	+ 74.41	+50	0	+ 200
GH-CNF10	5.0	+ 22.41	–	+ 104.65	+75	–	+ 600
GH-CNF15	2.0	+ 18.97	–	+ 44.19	+25	–	+ 100
EG-CNF1	3.0	+ 2.29	+6.81	+ 39.53	+25	0	+ 100
EG-CNF2	5.0	+ 3.45	+5.30	+ 40.70	0	0	0

## Conclusions

Morphology of the CNFs was influenced by production methods. The increase of pass numbers in microfluidizer facilitated the fibrillation degree of CNFs as well as reducing its diameter while endoglucanases hydrolysis enabled CNFs with smaller length. Moreover, CNF crystallinities was related to pass numbers in microfluidizer and enzymatic pretreatment. And transmittance of CNF films rose with smaller CNF widths distributions. Mechanical performance of the CNFs-reinforced paper was deeply affected by dosages, lengths, and types of CNFs. The GH-CNF10 with higher lengths exhibited better ability to improve tensile and burst strength of paper. The TE-CNF1 with smaller lengths and nematic network also displayed similar strengthened capabilities on paper as other CNFs. The improvement effect of CNFs on tensile index of paper increased as the CNF dosages rose from smaller amount up to 10.0 wt%. CNFs displayed worse enhancement effect on refined recycled pulp than unrefined hardwood pulp. However, change in burst index and folding endurance of CNFs-reinforced paper was contingent because of the complicated interaction between CNFs and the pulp fibers matrix. Folding endurance of paper was corresponded to fiber orientations. In general, GH-CNFs displayed better performance than EG-CNFs as paper strength additives. The most preeminent strengthened effect was found with GH-CNF10 with a dosage of 5.0 wt% on hardwood pulp, the tensile index, burst index, and folding endurance increased by 108.32%, 104.65%, and 600%, respectively.
